# Comprehensive preclinical evaluation of second-generation miRNA-regulated Coxsackievirus B3-BHP: Potent antitumor efficacy and safety

**DOI:** 10.1016/j.omton.2026.201332

**Published:** 2026-08-21

**Authors:** Yuki Sakashita, Shohei Miyamoto, Miyako Sagara, Shun Ito, Yukiko Kobayashi, Yoshio Momoda, Fumiaki Yano, Ken Eto, Kenzaburo Tani, Mutsunori Murahashi

**Affiliations:** 1 Division of Oncology, Research Center for Medical Sciences, The Jikei University School of Medicine, Tokyo 105-8461, Japan; 2 Department of Surgery, The Jikei University School of Medicine, Tokyo 105-8461, Japan; 3 Medical Center for Translational Research, Department of Medical Innovation, The University of Osaka Hospital, Osaka 565-0871, Japan; 4 Cell Processing Facilities, Research Center for Medical Sciences, The Jikei University School of Medicine, Tokyo 105-8461, Japan; 5 Department of Oncology and General Medicine, The Institute of Medical Science, The University of Tokyo, Tokyo 108-8639, Japan

**Keywords:** MT: Regular Issue, oncolytic virotherapy, CVB3, microRNA, preclinical study, biodistribution study, repeated-dose toxicity study

## Abstract

Oncolytic virotherapy employing Coxsackievirus B3 has emerged as a promising and innovative strategy for cancer treatment. However, wild-type Coxsackievirus B3 is associated with significant organ toxicities, such as viral hepatitis, myocarditis, and pancreatitis, which have presented major obstacles to its clinical application. In this study, we engineered a second-generation recombinant Coxsackievirus B3 (CVB3-BHP) by incorporating target sequences for the pancreas-specific miR-217 and the miR-34a, which is predominantly expressed in normal tissues, to further enhance its safety profile. Preclinical evaluation demonstrated that CVB3-BHP exhibited potent and dose-dependent antitumor efficacy in murine tumor models, with complete tumor regression or marked shrinkage observed in the high-dose group. Biodistribution analysis showed that CVB3-BHP transiently accumulated in the spleen and lacrimal gland but was rapidly cleared from these tissues over time, with limited excretion in urine and feces. Toxicity assessment indicated that even repeated high-dose administration did not induce overt systemic toxicity or organ damage. Although mild inflammation and acinar atrophy were observed in the lacrimal gland, no clinically significant adverse events were observed. Collectively, these findings indicate that CVB3-BHP combines robust antitumor efficacy with an improved safety profile, supporting its potential as a strong candidate for clinical translation in oncolytic virotherapy.

## Graphical abstract

**Figure:**
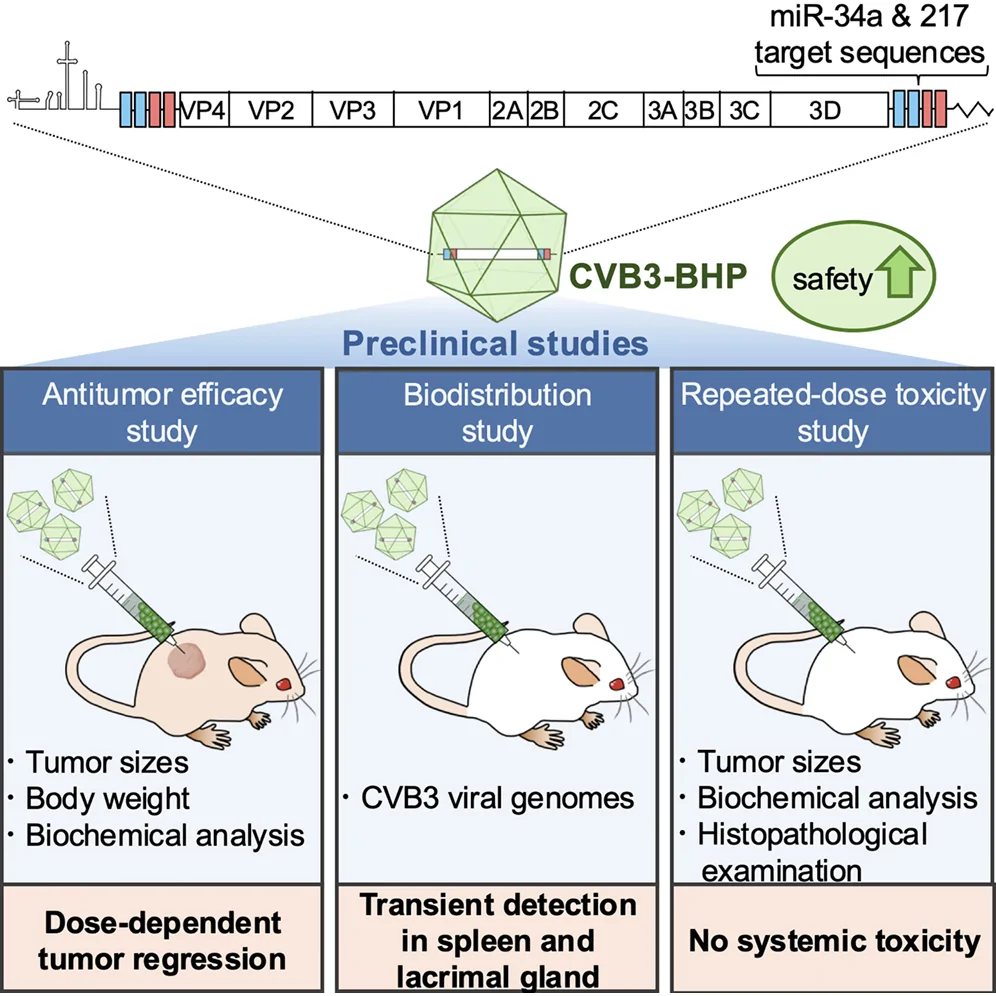

## Teaser

Murahashi and colleagues report that CVB3-BHP exhibits potent, dose-dependent antitumor efficacy in murine tumor models with reduced systemic and organ-specific toxicity, combining robust antitumor activity with an improved safety profile that supports its potential for clinical translation in oncolytic virotherapy.

## Introduction

Oncolytic virotherapy, which utilizes viruses that can selectively infect and lyse malignant cells, has gained increasing attention as an innovative cancer treatment strategy.^1^^,^^2^ This therapeutic modality ensures not only the direct destruction of tumor cells through viral replication but also the induction of potent antitumor immune responses. Virus-induced immunogenic cell death stimulates systemic antitumor immunity and is more likely to elicit robust immune responses compared with conventional necrosis or apoptosis. Various oncolytic viruses have been evaluated in clinical settings, including DNA viruses, such as adenovirus and herpes simplex virus (HSV), and RNA viruses, such as Coxsackievirus A21 (CVA21), measles virus, reovirus, and Newcastle disease virus.^3^^,^^4^^,^^5^^,^^6^ Among these, HSV and adenovirus have been particularly well-studied, owing to their broad applicability and therapeutic efficacy. Talimogene laherparepvec, a genetically modified HSV-1, was approved in 2015 by the US Food and Drug Administration (FDA) and the European Medicines Agency for the treatment of melanoma.^7^ In Japan, teserpaturev, a triple-mutated HSV-1, was approved by the Pharmaceuticals and Medical Devices Agency for malignant glioma in 2021.^8^ Further, in 2022, the FDA approved the recombinant adenovirus nadofaragene firadenovec-vncg, which encodes interferon-α2b, for use in bladder cancer therapy.^9^

Coxsackievirus, a member of the genus *Enterovirus* within the family Picornaviridae, is a non-enveloped, positive-sense, single-stranded RNA virus. Picornaviruses are attractive platforms for oncolytic virotherapy, owing to several unique characteristics. First, as RNA viruses, they replicate exclusively in the cytoplasm, thereby avoiding integration into the host genome and minimizing the risk of insertional mutagenesis. Second, their small, non-enveloped icosahedral capsid structure (approximately 25 nm in diameter) facilitates rapid replication and efficient delivery to target cells. Collectively, these properties have supported the clinical development of enterovirus-based oncolytic virotherapy in recent years. Previous studies have shown that CVA21 yields promising clinical outcomes in patients with non-muscle invasive bladder cancer and advanced malignant melanoma, whereas echovirus 1 has demonstrated efficacy in the treatment of ovarian cancer.^10^^,^^11^^,^^12^

Our previous studies demonstrated that CVB3 exhibits strong antitumor activity against various human cancer cells.^13^^,^^14^^,^^15^ However, the clinical application of wild-type (WT) CVB3 (CVB3-WT) is limited by its substantial systemic toxicities, including viral hepatitis, myocarditis, and pancreatitis. To address these concerns, Sagara et al. developed a modified virus (CVB3-HP) by inserting target sequences for the cardiac-specific miR-1 and the pancreatic-specific miR-217 into the 3′ untranslated region (UTR) of the CVB3 genome.^15^ This strategy exploited the RNA interference machinery to suppress viral replication in normal tissues, thereby reducing toxicity. CVB3-HP exhibited potent antitumor efficacy in a murine breast cancer xenograft model, comparable to that of CVB3-WT, while sparing exocrine pancreatic and cardiac tissues from damage, thereby achieving a favorable balance between efficacy and safety. Nonetheless, these findings were mainly derived from short-term intratumoral administration, and key issues—such as safety after repeated dosing, systemic distribution, viral excretion, and host immune responses—remain to be elucidated. Building upon this first-generation platform, we sought to enhance systemic safety while preserving tumor-selective replication. miR-217 was retained owing to its pancreas-enriched expression profile and its established role in limiting pancreatic toxicity.

In addition, we selected miR-34a based on its broad expression across normal tissues, including the heart and pancreas, and its frequent downregulation in multiple malignancies.^16^^,^^17^^,^^18^ miR-34a is a well-characterized p53-regulated tumor suppressor microRNA and has been shown to play functional roles in normal tissues, including the regulation of cardiac physiology.^16^^,^^18^^,^^19^ Consistent with these properties, our previous study demonstrated that miR-34a is abundantly expressed in normal cardiac and pancreatic tissues, while exhibiting reduced expression in several tumor types, including lung cancer. Furthermore, miR-34a-mediated targeting effectively attenuated CVB3 pathogenicity without compromising antitumor activity.^14^ Together, these findings support the feasibility of miR-34a as a broadly acting safety regulator that suppresses viral replication in normal tissues while permitting tumor-selective viral propagation. In contrast, although miR-1 is highly expressed in cardiac muscle and was used as a cardiac safety regulator in the first-generation construct, its expression is largely restricted to muscle tissues and, therefore, provides limited protection beyond the heart.^15^ In this second-generation design, miR-34a was introduced in place of miR-1 to enable more comprehensive regulation of viral replication across normal tissues, while maintaining effective cardiac safety control.

Based on this design strategy, we generated a second-generation recombinant CVB3 (CVB3-BHP) and conducted a comprehensive preclinical evaluation. This included analyses of antitumor efficacy, biodistribution, excretion, and toxicity following repeated administration. As a next-stage preclinical study building upon our prior work, these investigations advance the rational development of microRNA-regulated enteroviral oncolytic platforms and support the potential clinical translation of safer CVB3-based virotherapy.

## Results

### CVB3-BHP demonstrated reduced blood toxicity while retaining antitumor activity

**Figure 1:**
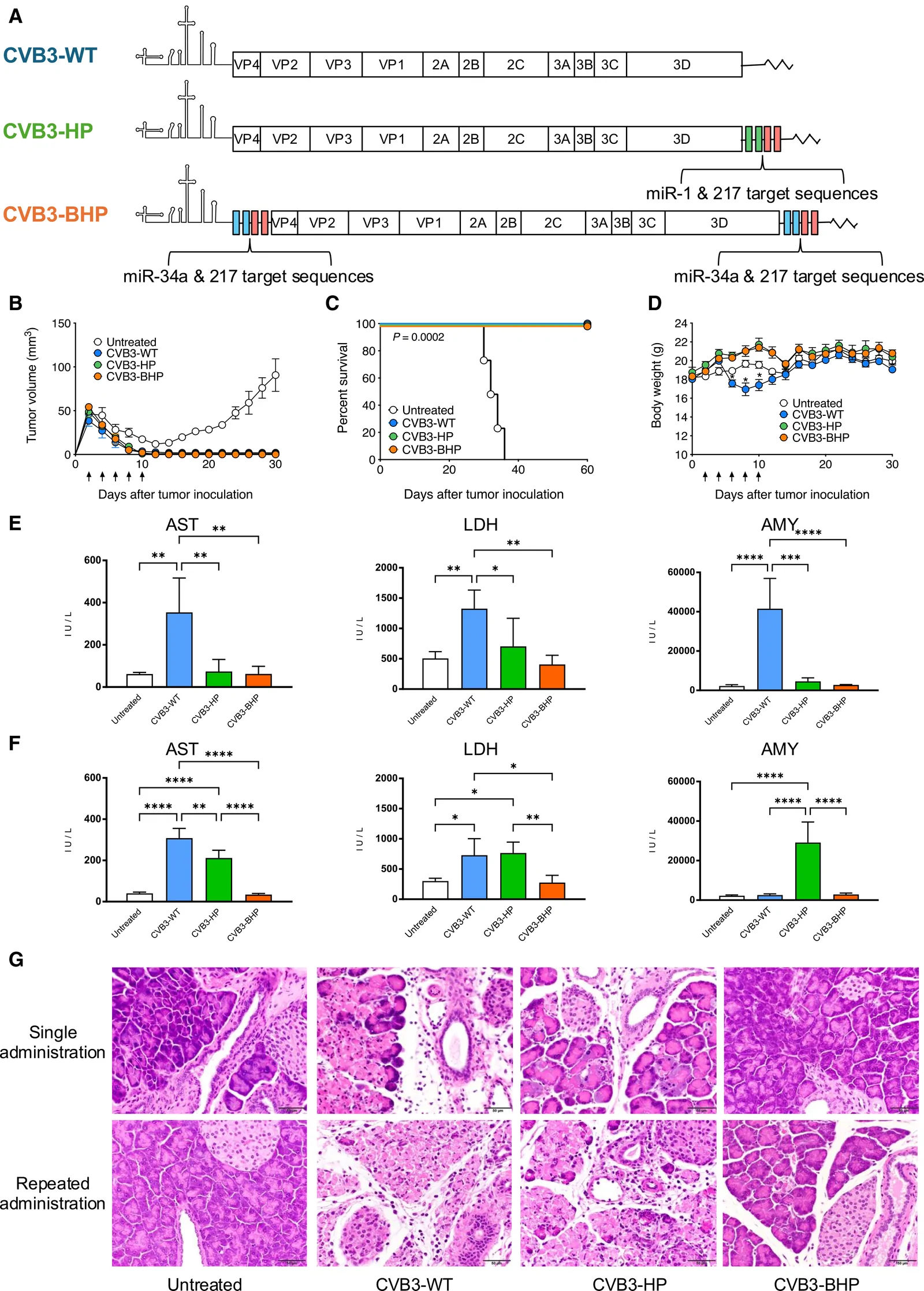
Antitumor efficacy and safety profile of CVB3-BHP (A) Genomic structure. Schematic representation of the CVB3-BHP genome. Target sequences for miR-217 and miR-34a were inserted into the 5′ and 3′ untranslated regions (UTRs), respectively. (B–D) *In vivo* evaluation in a non-small cell lung cancer xenograft model. H1299 tumor-bearing BALB/c-*nu*/*nu* mice (*n* = 5 per group) received intratumoral administration of CVB3-WT, CVB3-HP, CVB3-BHP (5 × 10^6^ TCID_50_/mouse), or saline. Tumor volumes and body weights were measured every other day. (B) Tumor volume curves for each group. (C) Kaplan-Meier survival curves. (D) Body weight changes during the experimental period. (E) Serum biochemistry after a single administration. Serum biochemical parameters measured one day after the first administration (*n* = 4 per group). (F) Serum biochemistry after two administrations. Serum biochemical parameters measured one day after the second administration (*n* = 4 per group). (G) Pancreatic histopathology. Representative hematoxylin and eosin (H&E) staining of pancreatic tissue from untreated control and virus-treated groups (*n* = 4 per group). Upper images show findings after a single administration, and lower images show findings after two administrations. Scale bars, 50 μm. Data are presented as mean (SD). ∗*p* < 0.05; ∗∗*p* < 0.01; ∗∗∗*p* < 0.001; ∗∗∗∗*p* < 0.0001. AMY, amylase; AST, aspartate aminotransferase; CVB3-BHP, second-generation recombinant Coxsackievirus B3; CVB3-HP, first-generation recombinant Coxsackievirus B3; CVB3-WT, wild-type Coxsackievirus B3; LDH, lactate dehydrogenase; SD, standard deviation; TCID_50_, tissue culture infectious dose 50%.

The genomic structures of the viruses used in this study are schematically presented in Figure 1A. Three variants were compared: CVB3-WT, the previously reported first-generation recombinant virus (CVB3-HP), and the second-generation recombinant virus (CVB3-BHP).

In the H1299 xenograft nude mouse model, CVB3-BHP induced potent tumor regression, comparable to that achieved with CVB3-WT and CVB3-HP (Figure 1B). All the virus-treated groups exhibited significantly prolonged survival relative to untreated controls (Figure 1C). Monitoring of body weight revealed transient weight loss in only the CVB3-WT group, whereas the CVB3-HP and CVB3-BHP groups maintained stable weights comparable to those of untreated mice (Figure 1D). These findings indicate that both CVB3-HP and CVB3-BHP result in markedly lower systemic adverse effects than those observed with CVB3-WT. Serum biochemical analyses after a single administration revealed pronounced elevations in aspartate aminotransferase (AST), lactate dehydrogenase (LDH), and amylase (AMY) levels in the CVB3-WT group (Figure 1E). The CVB3-HP group also showed an increase in these parameters, although to a lesser extent than did CVB3-WT, indicating partial reduction of blood toxicity. In contrast, the CVB3-BHP group showed no such elevations, with values comparable to those of controls. After two administrations, the CVB3-HP group continued to exhibit increases in AST, LDH, and AMY levels (Figure 1F). In the CVB3-WT group, AST and LDH levels remained elevated, but AMY levels did not increase. This lack of AMY elevation likely reflects severe pancreatic injury induced by CVB3-WT, leading to a loss of AMY-producing capacity. Notably, the CVB3-BHP group showed no biochemical abnormalities even after repeated dosing, with the results similar to those of untreated controls (Figure 1F). Histopathological analysis of the pancreas further substantiated these biochemical findings (Figure 1G). After a single administration, the CVB3-WT group exhibited extensive coagulative necrosis of pancreatic acinar cells, whereas the CVB3-HP group showed evident but less severe acinar injury. In contrast, the CVB3-BHP group preserved normal acinar architecture comparable to that of untreated controls. Following two administrations, pancreatic damage in the CVB3-WT group progressed to near-complete destruction of acinar structures. The CVB3-HP group also developed widespread acinar necrosis after repeated dosing, although the overall severity remained lower than that observed in the CVB3-WT group. Consistent with these histopathological changes, serum AMY levels in the CVB3-WT group failed to increase after the second administration, consistent with extensive acinar cell loss and consequent depletion of AMY-producing capacity. In contrast, CVB3-BHP caused no discernible pancreatic injury even after repeated dosing. Collectively, these findings demonstrate a clear, stepwise attenuation of pancreatic toxicity from CVB3-WT to CVB3-HP and further to CVB3-BHP, fully concordant with the observed serum biochemical profiles.

### CVB3-BHP demonstrated dose-dependent antitumor efficacy without inducing detectable toxicity

Building on the promising antitumor efficacy and reduced hematological toxicity of CVB3-BHP observed in the H1299 xenograft model, we conducted comprehensive non-clinical studies in MDA-MB-468 tumor-bearing BALB/c-*nu*/*nu* mice to further evaluate its therapeutic potential and safety. For scientific rigor and to minimize animal use, only saline control and CVB3-BHP treatment groups at varying doses were included; CVB3-WT and CVB3-HP were not tested in this series of experiments.

**Figure 2:**
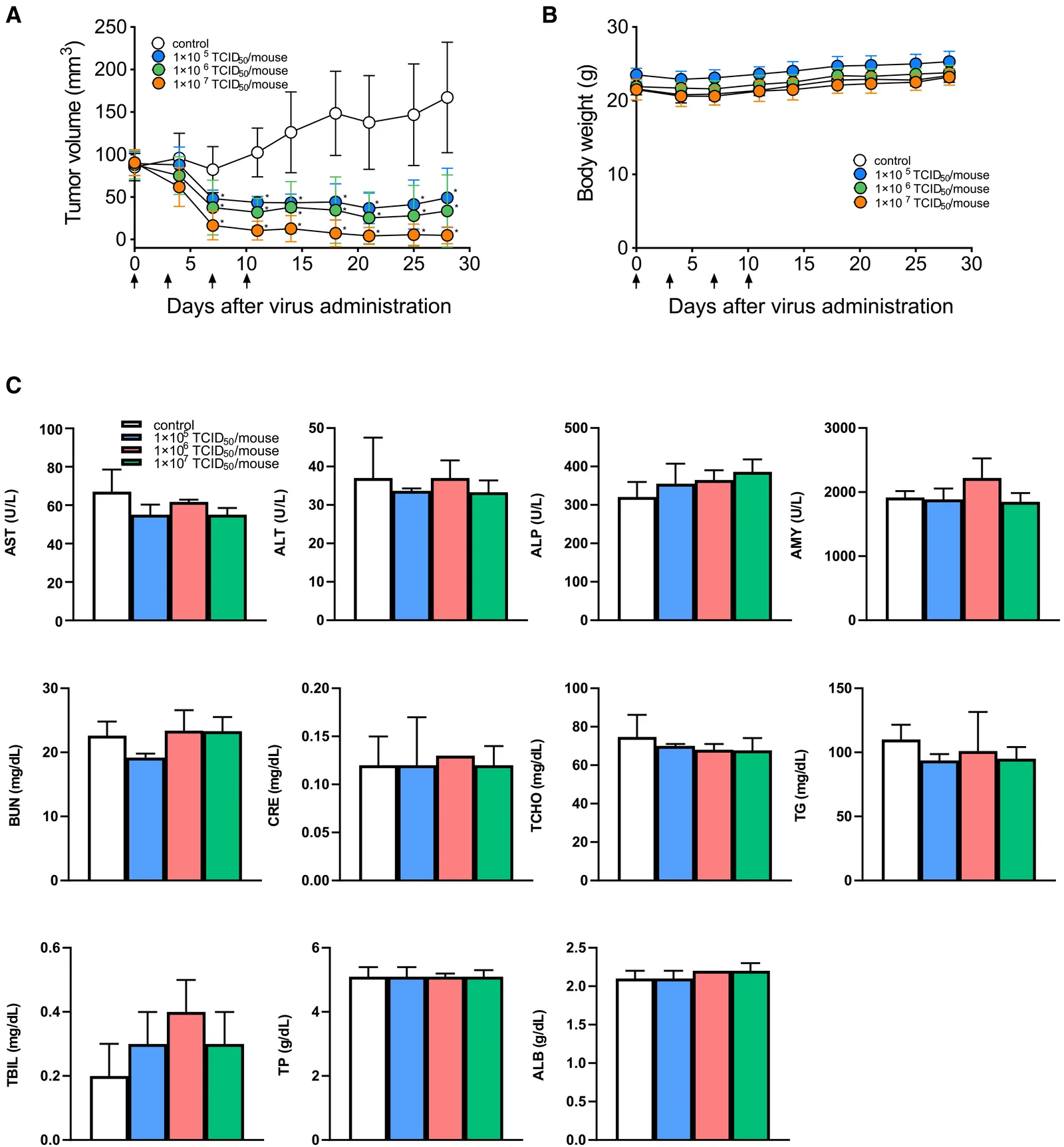
Antitumor efficacy and safety of intratumoral CVB3-BHP (A) Tumor growth. Tumor volume after intratumoral administration of CVB3-BHP in MDA-MB-468 tumor-bearing BALB/c-*nu*/*nu* mice (*n* = 6 per group). Mice were treated on days 0, 3, 7, and 10 with three different doses. (B) Body weight. Body weight changes during the experimental period (*n* = 6 per group). (C) Serum biochemistry. Serum biochemical parameters measured at necropsy (*n* = 3 per group). Data are presented as mean (SD). ∗*p* < 0.05 vs. control. ALB, albumin; ALP, alkaline phosphatase; ALT, alanine aminotransferase; AMY, amylase; AST, aspartate aminotransferase; BUN, blood urea nitrogen; CRE, creatinine; CVB3-BHP, second-generation recombinant Coxsackievirus B3; TBIL, total bilirubin; TCHO, total cholesterol; TCID_50_, tissue culture infectious dose 50%; TG, triglycerides; TP, total protein.

All the CVB3-BHP-treated groups exhibited significant tumor growth suppression compared with that in controls, beginning on day 7 after the first administration (Figure 2A). Tumor volume reduction was dose-dependent, with the high-dose group (1.0 × 10^7^ tissue culture infectious dose 50% [TCID_50_]/mouse) achieving complete tumor regression in 5 of 6 mice after day 18, underscoring the potent antitumor activity of CVB3-BHP. Body weights remained stable in all groups throughout the study, with no significant differences between the treated and control mice during the experimental period or at the endpoint (Figure 2B). This stability mirrors prior *in vivo* findings in which CVB3-WT administration induced weight loss, whereas CVB3-BHP did not, regardless of dose. In addition, no systemic adverse effects were observed following CVB3-BHP administration. Serum biochemical analyses showed no abnormal elevations in any CVB3-BHP-treated group (Figure 2C). All values remained within physiological ranges and were comparable to those of untreated controls.

### CVB3-BHP exhibited transient biodistribution, primarily in the spleen and lacrimal gland, with rapid clearance

Here, we employed quantitative real-time polymerase chain reaction (PCR) to examine the temporal and spatial distribution of CVB3-BHP genomes in multiple organs and excreta of male and female mice after administration.

**Figure 3:**
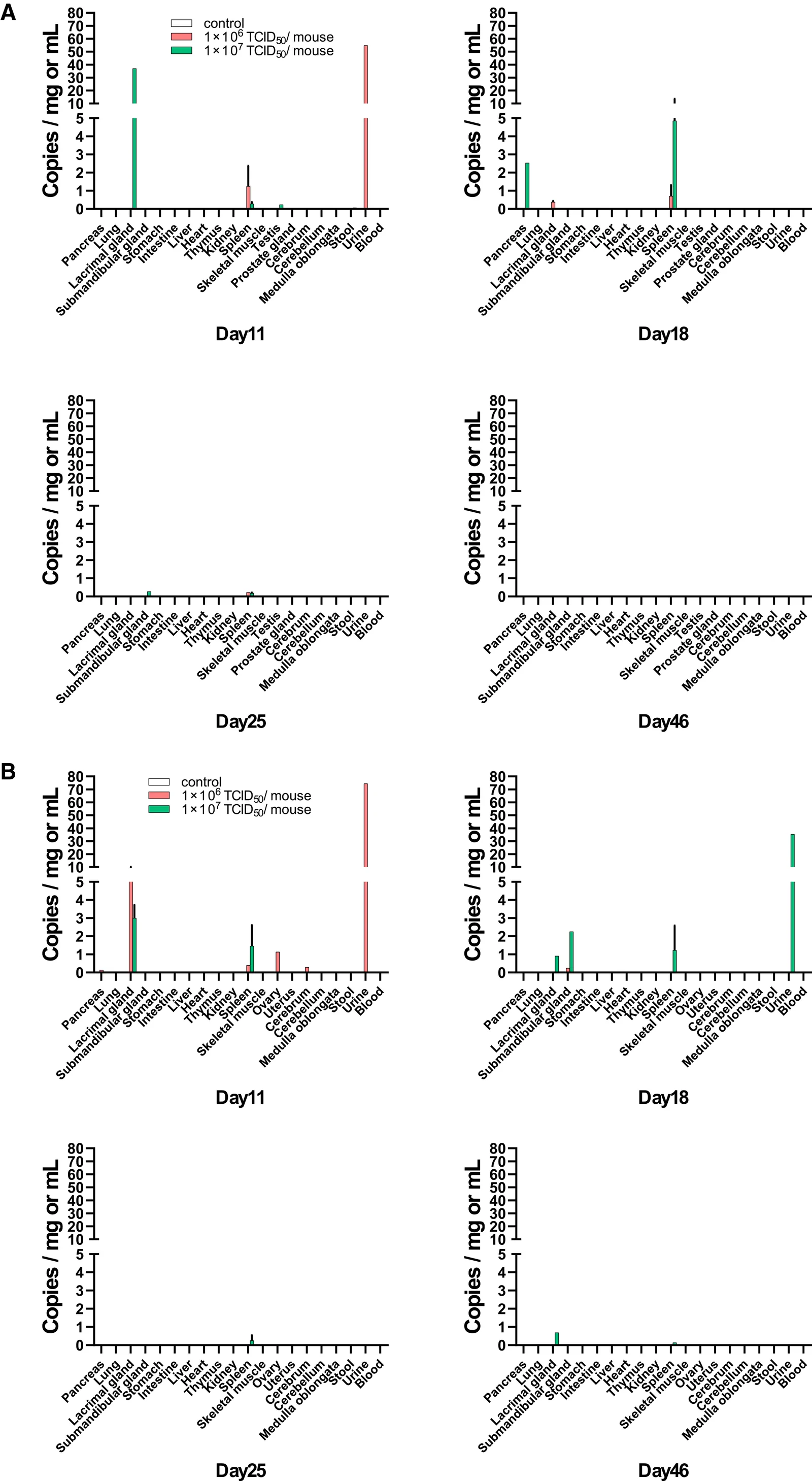
Biodistribution of CVB3-BHP after repeated subcutaneous administration (A) Male mice. Viral RNA copy numbers in organs and body fluids from male Crl:CD1 (ICR) mice (*n* = 4 per group) on days 11, 18, 25, and 46 following subcutaneous administration of 1.0 × 10^6^ or 1.0 × 10^7^ TCID_50_/mouse on days 0, 3, 7, and 10. Viral RNA was quantified using real-time PCR and is shown as copies per mg of tissue or per mL of fluid (log scale). (B) Female mice. Viral RNA copy numbers in organs and body fluids from female mice (*n* = 4 per group) analyzed under the same conditions. Values below the detection limit are shown as 0. CVB3-BHP, second-generation recombinant Coxsackievirus B3; PCR, polymerase chain reaction; TCID_50_, tissue culture infectious dose 50%.

In male mice, CVB3-BHP genomes were predominantly detected in the spleen and the lacrimal gland on day 11 post-administration, which corresponds to the day after the final viral administration (Figure 3A). Viral genomes persisted in the spleen through day 18 but were largely undetectable by day 25, indicating nearly complete clearance. In the lacrimal gland, viral genomes were present from day 11 to day 18 and undetectable by day 25. Female mice exhibited a similar pattern (Figure 3B), although detection in the lacrimal gland was slightly more frequent than that in males. By day 25, viral genomes were absent or near the detection limit in all tissues in both sexes. Excretion analysis revealed transient detection of viral genomes in urine between days 11 and 18 in both sexes, with no detectable virus beyond day 25. No substantial sex-related differences were observed in the distribution or clearance kinetics. In other tissues—including the testes, pancreas, submandibular gland, brain, ovaries, and feces—viral genomes were detected only sporadically at low levels, with no evidence of sustained accumulation at any time point.

### Repeated subcutaneous administration of CVB3-BHP caused only limited histopathological changes in the lacrimal gland and the spleen with no systemic toxicity

**Figure 4:**
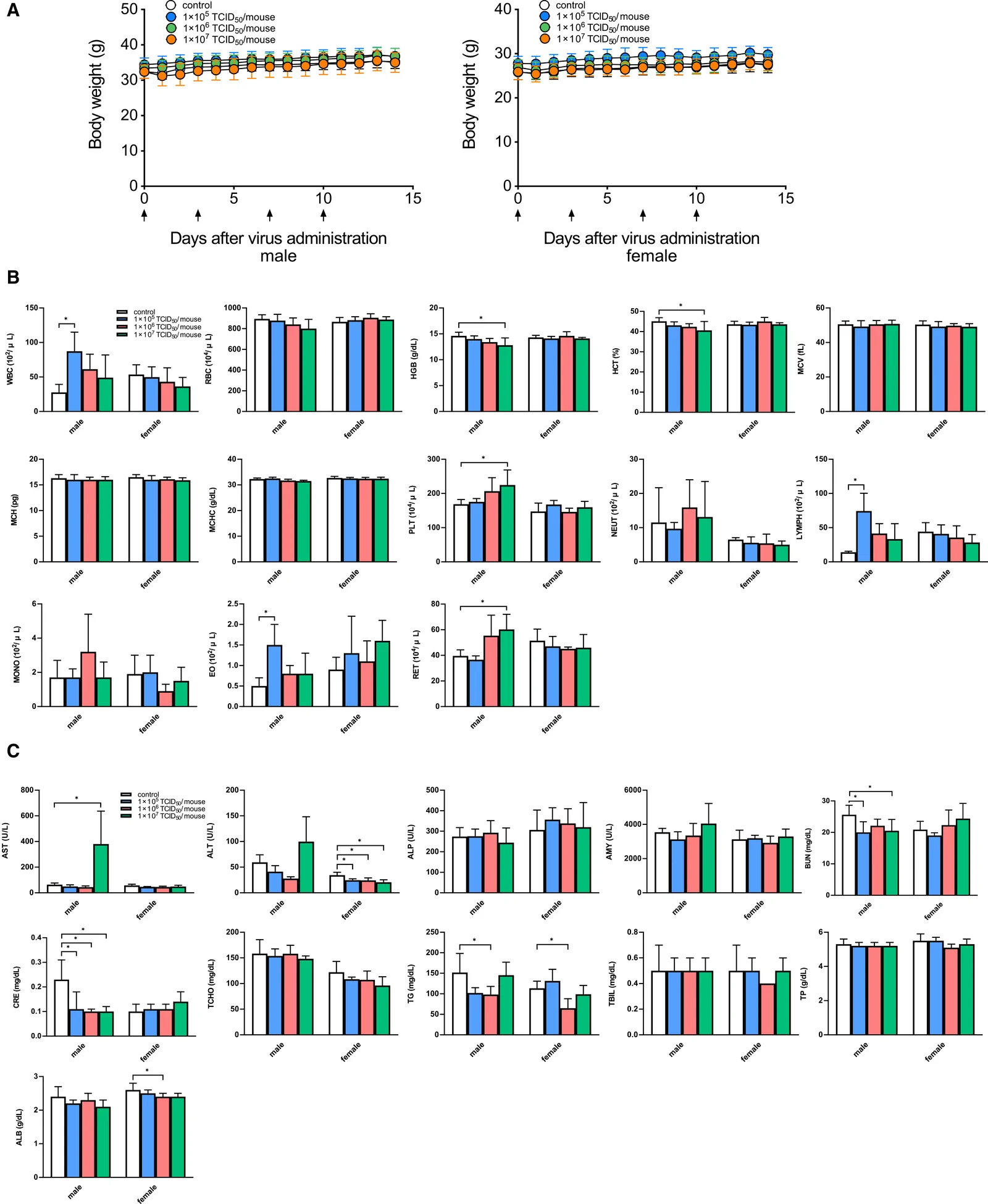
Toxicological evaluation of repeated subcutaneous CVB3-BHP administration (toxicity groups) (A) Body weight. Body weight changes in male (left) and female (right) Crl:CD1 (ICR) mice (*n* = 10 per group) receiving 1.0 × 10^5^, 1.0 × 10^6^, or 1.0 × 10^7^ TCID_50_/mouse on days 0, 3, 7, and 10. (B) Hematological parameters. Hematological analysis performed on day 14 (*n* = 10 per group). Basophils were not detected in the hematological analyses and are, therefore, not shown in the figure. (C) Serum biochemistry. Serum biochemical parameters measured on day 14 (*n* = 10 per group). Data are presented as mean (SD). ∗*p* < 0.05 vs. control. ALB, albumin; ALP, alkaline phosphatase; ALT, alanine aminotransferase; AMY, amylase; AST, aspartate aminotransferase; BUN, blood urea nitrogen; CRE, creatinine; CVB3-BHP, second-generation recombinant Coxsackievirus B3; EO, eosinophil count; HCT, hematocrit; HGB, hemoglobin; LYMPH, lymphocyte count; MCH, mean corpuscular hemoglobin; MCHC, mean corpuscular hemoglobin concentration; MCV, mean corpuscular volume; MONO, monocyte count; NEUT, neutrophil count; PLT, platelet count; RBC, red blood cell count; RET, reticulocyte count; TBIL, total bilirubin; TCHO, total cholesterol; TCID_50_, tissue culture infectious dose 50%; TG, triglycerides; TP, total protein; WBC, white blood cell count.

**Figure 5:**
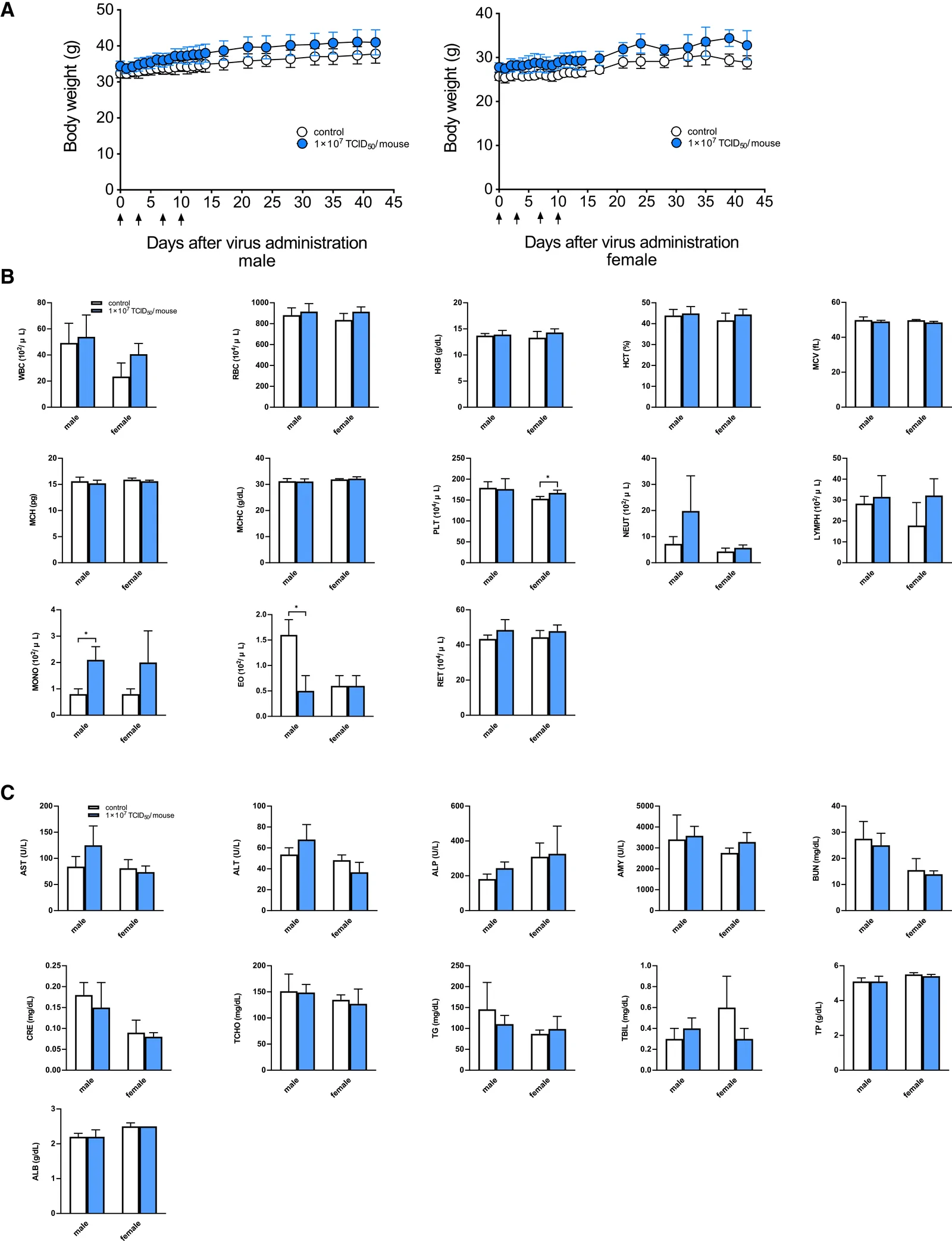
Toxicological evaluation of repeated subcutaneous CVB3-BHP administration (recovery groups) (A) Body weight. Body weight changes in recovery groups (*n* = 6 per group) following administration of 1.0 × 10^7^ TCID_50_/mouse. (B) Hematological parameters. Hematological analysis performed on day 42 (*n* = 6 per group). Basophils were not detected in the hematological analyses and are, therefore, not shown in the figure. (C) Serum biochemistry. Serum biochemical parameters measured on day 42 (*n* = 6 per group). Data are presented as mean (SD). ∗*p* < 0.05 vs. control. ALB, albumin; ALP, alkaline phosphatase; ALT, alanine aminotransferase; AMY, amylase; AST, aspartate aminotransferase; BUN, blood urea nitrogen; CRE, creatinine; CVB3-BHP, second-generation recombinant Coxsackievirus B3; EO, eosinophil count; HCT, hematocrit; HGB, hemoglobin; LYMPH, lymphocyte count; MCH, mean corpuscular hemoglobin; MCHC, mean corpuscular hemoglobin concentration; MCV, mean corpuscular volume; MONO, monocyte count; NEUT, neutrophil count; PLT, platelet count; RBC, red blood cell count; RET, reticulocyte count; TBIL, total bilirubin; TCHO, total cholesterol; TCID_50_, tissue culture infectious dose 50%; TG, triglycerides; TP, total protein; WBC, white blood cell count.

To evaluate the reversibility of any toxic effects, the study incorporated a “toxicity group” (Figure 4) necropsied immediately after the final administration and a “recovery group” (Figure 5) examined following a defined post-treatment period.

General health parameters—including body weight changes (Figures 4A and 5A), urinalysis, food intake, and ophthalmologic assessments (Table S1)—were monitored in both sexes throughout the study. No significant abnormalities were detected in any group.

Hematological evaluation showed that, in males, the low-dose toxicity group exhibited significantly increased white blood cell count (WBC), lymphocyte count (LYMPH), and eosinophil count (EO) compared with those of controls; however, they were attributable to a single mouse exhibiting high level counts and lacked dose dependency, leading us to consider them incidental (Figure 4B). In the high-dose group, a significant decrease in hemoglobin (HGB) and hematocrit (HCT) was observed, whereas a significant increase in platelet count (PLT) and reticulocyte count (RET) was noted. In females, no significant hematological changes were detected at any dose. Notably, the significant changes observed in high-dose males were absent during the recovery phase, whereas increased monocyte count (MONO) and decreased EO were noted in males, and an increase in PLT was observed in females (Figure 5B).

With respect to serum biochemistry, males in the toxicity groups demonstrated significant changes in AST, blood urea nitrogen (BUN), creatinine (CRE), and triglyceride (TG) levels. In females, significant decreases were observed in alanine aminotransferase (ALT) levels at all doses as well as in TG and albumin (ALB) levels in the middle-dose group (Figure 4C). At recovery, none of these significant changes were present in either sex, indicating that they were transient (Figure 5C). Overall, the hematological and biochemical changes were minor and reversible during the recovery phase.

Histopathological examination identified changes primarily in the lacrimal gland and the spleen attributable to CVB3-BHP administration (Table S2). In the lacrimal glands, diffuse mononuclear cell infiltration and acinar atrophy occurred in all treatment groups, regardless of sex, with more pronounced atrophy in female mice and persistence into the recovery phase in some cases (Table S3). In the spleen, extramedullary hematopoiesis was observed in male mice receiving medium or high doses but had resolved completely by the recovery assessment. Inflammatory cell infiltration was also noted at injection sites, interpreted as a local reaction to the administration procedure. All other incidental findings were considered spontaneous background lesions unrelated to CVB3-BHP treatment or sex.

## Discussion

In this study, we comprehensively evaluated the antitumor efficacy, safety, biodistribution, and excretion profile of CVB3-BHP, a second-generation recombinant CVB3. CVB3-BHP was specifically designed to reduce the toxicity of the first-generation recombinant CVB3-HP, which contains target sequences for pancreas-specific miR-217 and myocardium-specific miR-1. To enhance tumor selectivity and reduce off-target effects, the miR-1 target sequence used in the first-generation construct was replaced with a target sequence for miR-34a, a microRNA widely expressed in normal tissues but frequently downregulated in diverse malignancies.^16^^,^^17^ In the MDA-MB-468 tumor-bearing mouse model, repeated intratumoral administration of CVB3-BHP produced a clear dose-dependent suppression of tumor growth and complete regression in several mice in the high-dose group. Notably, compared with CVB3-WT, CVB3-BHP maintained a strong antitumor activity while avoiding the transient weight loss and serum biochemical abnormalities typically associated with the WT virus. Biodistribution analysis showed that CVB3-BHP exhibited transient localization predominantly in the spleen and the lacrimal glands, with viral genomes cleared from nearly all tissues by day 25. In a few female mice, trace amounts of viral genomes were detectable in the lacrimal glands at day 46; however, these levels were close to the assay detection limit and indicated negligible long-term persistence. Viral genomes were rarely detected in urine or feces, suggesting minimal risk of environmental shedding. Few previous studies have systematically evaluated the safety of repeated or systemic dosing in oncolytic virotherapy. In this context, our work employed both repeated intratumoral and subcutaneous administration, which are clinically feasible routes, and generated a detailed dataset encompassing systemic safety, biodistribution, and excretion kinetics. These results provide a strong preclinical basis for the future clinical development of CVB3-BHP. It should be noted that the therapeutic performance of miRNA-regulated oncolytic viruses may depend on the endogenous miRNA landscape of the target tumor. Although miR-34a is frequently downregulated in many cancers, its expression levels can vary among tumor types and even among molecular subtypes of the same malignancy.^16^^,^^17^ Consequently, tumors that retain relatively high levels of these miRNAs may exhibit reduced viral replication and potentially attenuated antitumor efficacy.

In light of this variability, while tumor- or patient-specific optimization of miRNA target sequences represents a theoretically attractive strategy, the complexity and heterogeneity of miRNA expression across different cancer types raise substantial concerns regarding both safety and feasibility. Therefore, at present, a more practical approach is to systematically investigate the range of endogenous miRNA expression levels that permit replication of miRNA-regulated viruses and to better understand how endogenous miRNA profiles influence the balance between safety and antitumor efficacy across diverse tumor contexts. Such studies may also establish endogenous miRNA expression profiles as predictive biomarkers for therapeutic efficacy and help identify patients most likely to benefit from CVB3-BHP therapy. In the future, pretreatment assessment of tumor miRNA expression profiles, including miR-34a and miR-217, may enable a personalized treatment strategy by identifying patients whose tumors are expected to support efficient viral replication and respond favorably to CVB3-BHP therapy. This knowledge will facilitate appropriate patient selection for CVB3-BHP therapy.

Regarding biodistribution, the spleen and the lacrimal glands were the main organs in which viral genomes were detected. Both tissues are known to express the Coxsackievirus and adenovirus receptor (CAR), the major receptor for Coxsackievirus and adenovirus.^20^ CAR expression in exocrine glands, including the ductal and acinar cells of rat lacrimal glands, has been demonstrated in animal studies.^21^ In CVB3, infection and replication following CAR-mediated cellular entry are considered the principal mechanisms of infection. Accordingly, viral presence in tears is presumed to result predominantly from release by infected cells. In contrast, Hsueh et al. reported a CAR-dependent transcytosis pathway that enables blood-derived substances to be internalized and secreted toward the luminal side.^22^ This transport mechanism suggests a potential route by which circulating particles may traverse lacrimal gland epithelial cells and reach the luminal compartment. Therefore, although a fraction of circulating viral particles may be transported to the luminal side via this transcytosis pathway, viral appearance in tears is likely to be mainly attributable to release from infected cells. Taken together, the viral detection, mononuclear cell infiltration, and acinar atrophy observed in the lacrimal glands in this study are most likely attributable to the combined effects of CAR-mediated viral entry and replication, together with lacrimal gland-specific secretory and transport mechanisms.

In addition, suppression of viral replication in the lacrimal gland may have been less effective than that in other tissues. CVB3-BHP was designed to improve tumor selectivity and control systemic toxicity by incorporating a target sequence for miR-34a. However, little is known about miRNA expression in the lacrimal gland. In a rabbit model of autoimmune dacryoadenitis, miR-34a expression was markedly reduced, but data for normal mouse or human lacrimal glands remain limited.^23^ Therefore, determining the contribution of miRNA target sequences to replication control in lacrimal glands should be a future research priority. The viral accumulation and histopathological injury observed in this study suggest that miRNA-mediated suppression in the lacrimal gland was not fully effective. In addition, lacrimal gland acinar cells have limited regenerative capacity, and irreversible structural damage can persist after severe or chronic injury, as demonstrated in mouse models.^24^^,^^25^ The injury observed in this study can, therefore, be interpreted as a consequence of CAR-mediated viral entry and accumulation, incomplete miRNA-based replication control, and damage exceeding the intrinsic repair capacity of the gland. Future optimization of the miRNA-targeting strategy, including the incorporation of target sequences for lacrimal gland-enriched miRNAs, may further reduce off-target viral replication, thereby mitigating lacrimal gland inflammation and acinar atrophy while preserving antitumor efficacy.

In the spleen, viral detection most likely reflects transient accumulation of circulating viral particles rather than sustained local replication. This is likely attributable to transient detection of viral genomes within immune cells following the uptake of circulating viral particles. In addition, the incorporation of miRNA target sequences may have contributed, at least in part, to limiting viral replication within splenic tissue. The extramedullary hematopoiesis observed in this study can most plausibly be explained by immune activation in response to temporary antigenic stimulation by viral particles. While miRNA regulation can suppress replication within tissues, it may not completely prevent the accumulation of viral particles from the bloodstream or antigen presentation, and some degree of immune activation is, therefore, unavoidable. Sex-related differences in extramedullary hematopoiesis also merit consideration. Prior studies of CVB3 infection have shown that sex can influence viral susceptibility and immune responses. Female mice generate stronger splenic CD8^+^ T cell responses and achieve greater viral clearance, whereas male mice are more susceptible to severe infection, in part due to the immunosuppressive effects of testosterone.^26^^,^^27^ The differences in extramedullary hematopoiesis, serum biochemical findings, and viral distribution observed here may, therefore, reflect underlying hormonal and immunological differences between sexes.

In the repeated-dose toxicity testing, no severe toxicity attributable to CVB3-BHP was observed in the clinical condition, body weight, food consumption, hematology, serum biochemistry, urinalysis, or ophthalmologic examination. In this study, doses up to 1.0 × 10^7^ TCID_50_ per body were evaluated; however, the maximum tolerated dose (MTD) was not reached within this range, indicating a sufficiently wide safety margin below the MTD. Oncolytic viruses have recently attracted attention for their ability to act synergistically with immune checkpoint inhibitors.^28^^,^^29^ A systemically administrable virus, such as CVB3-BHP, combines high tumor selectivity with the potential to target disseminated metastases and multiple primary tumors, thereby expanding therapeutic options. Moreover, the miRNA-based regulatory strategy could be applied to other viral platforms and cancer types, making it a versatile approach for the design of oncolytic viruses.

This study has several limitations that should be addressed in future investigations. First, as the biodistribution and toxicity studies were conducted in murine models, interspecies differences in immune responses and tissue tropism may affect the translation of these findings to humans. Second, while the incorporation of miR-217 and miR-34a target sequences successfully improved the safety profile of CVB3-BHP, the efficacy of miRNA-mediated replication control in human lacrimal glands and other exocrine tissues remains uncertain, owing to the limited availability of data on their endogenous miRNA expression patterns. Third, although our repeated-dose toxicity study demonstrated a wide safety margin at doses up to 1.0 × 10^7^ TCID_50_, the MTD was not reached, necessitating further evaluation of higher doses to fully define the therapeutic window. Fourth, systemic toxicity following intravenous administration was not evaluated. Intravenous delivery could potentially expose a broader range of normal tissues to circulating viral particles and may, therefore, reveal higher levels of systemic toxicity than with local administration routes such as intratumoral or subcutaneous injection. Importantly, in the present study, we performed repeated-dose toxicity assessments across a wide dose range, including doses substantially exceeding the anticipated therapeutic levels. Although this approach does not fully replicate intravenous administration, it enabled us to evaluate the systemic biodistribution and toxicity of CVB3-BHP under repeated high-dose conditions. Indeed, biodistribution analyses demonstrated that, even after subcutaneous administration, viral genomes were transiently detectable in systemic organs, such as the spleen and the lacrimal glands, indicating that the virus can disseminate beyond the injection site. Despite this systemic exposure, no significant treatment-related toxicity was observed. Taken together, the findings of this study provide preliminary evidence supporting the systemic safety of CVB3-BHP. Nevertheless, dedicated intravenous administration studies are required to fully characterize its systemic safety profile before clinical translation.

In addition, antitumor efficacy in this study was evaluated using a xenograft model in immunodeficient mice to assess the intrinsic oncolytic activity of CVB3-BHP while minimizing the confounding effects of adaptive immunity. In immunocompetent hosts, antiviral immune responses can promote rapid viral clearance, which may reduce intratumoral viral titers and consequently attenuate direct oncolytic activity. Indeed, previous studies have demonstrated that host antiviral immunity can limit viral persistence and thereby reduce therapeutic efficacy in certain contexts. At the same time, oncolytic virotherapy is known to induce immunogenic cell death and stimulate antitumor immune responses, which can contribute to therapeutic efficacy beyond direct viral oncolysis. Therefore, although viral persistence may be reduced in immunocompetent settings, the overall antitumor effect is likely determined by a balance between antiviral clearance and immune-mediated antitumor activity. Notably, CVB3-BHP may exhibit reduced viral loads *in vivo* because of its attenuated replication in normal tissues, which could potentially lead to more rapid viral clearance compared with WT and CVB3-HP strains. On the other hand, reduced off-target viral replication may also attenuate systemic antiviral immune activation, which could influence viral clearance kinetics in a more complex manner. Consistent with these considerations, we evaluated CVB3-BHP in an immunocompetent TC-1 syngeneic tumor model and demonstrated that CVB3-BHP retained significant antitumor efficacy in C57BL/6 mice (Figure S1). These findings support the therapeutic potential of CVB3-BHP in the presence of an intact host immune system. Nevertheless, further studies are warranted to clarify how host immune responses influence viral clearance, antitumor immunity, and the durability of therapeutic efficacy. Lastly, while immunocompetent mice, such as Crl:CD1 (ICR), were used for biodistribution and toxicity assessments, these preclinical models cannot fully capture the complexity of human immune responses. Future studies should further clarify how host immunity influences viral clearance, antitumor responses, and systemic safety by using clinically relevant immunocompetent models along with detailed immunological analyses.

Given that long-term persistence of replicating virus is neither desirable nor likely to be feasible in immunocompetent hosts, future therapeutic strategies should focus on enhancing durable antitumor immunity following viral clearance rather than prolonging viral persistence. In this regard, incorporation of immunostimulatory cytokines or other immune-modulating transgenes into CVB3-BHP may represent a promising approach to strengthen antitumor immune responses, reduce the risk of tumor recurrence, and improve the durability of therapeutic efficacy. Future work should also focus on incorporating additional miRNA target sequences to enhance the control of lacrimal gland infection, optimizing manufacturing processes, and conducting rigorous preclinical safety evaluations to support the initiation of clinical trials.

Taken together, the findings of this study suggest that CVB3-BHP is a promising candidate for clinical development as an oncolytic virus, providing enhanced tumor selectivity and reduced systemic toxicity through miRNA-mediated regulation.

## Methods

### Cell lines

The human non-small cell lung cancer cell line, NCI-H1299 (H1299), and the human breast cancer cell line, —MDA-MB-468, were obtained from the American Type Culture Collection (Manassas, VA, USA). The H1299-T7 cell line was generated by introducing the T7 RNA polymerase gene into parental H1299 cells via a lentiviral vector. H1299 and H1299-T7 cells were maintained in RPMI 1640 medium (Nacalai Tesque, Kyoto, Japan) supplemented with 10% fetal bovine serum (FBS; Thermo Fisher Scientific K.K., Tokyo, Japan) and 1% penicillin-streptomycin solution (FUJIFILM Wako Pure Chemical Corporation, Osaka, Japan). MDA-MB-468 cells were maintained in Leibovitz’s L-15 medium (Thermo Fisher Scientific K.K., Tokyo, Japan) with 10% FBS and 1% penicillin-streptomycin solution. All cell lines were incubated at 37°C in a humidified atmosphere containing 5% CO_2_.

### Mice

Six-week-old female BALB/c-*nu*/*nu* mice (Charles River Laboratories Japan, Inc., Kanagawa, Japan) were sacrificed for *in vivo* experiments using the H1299 cell line. In the antitumor efficacy studies, five-week-old female BALB/c-*nu*/*nu* mice from the same supplier were used. For the biodistribution study, five-week-old male and female Crl:CD1 (ICR) mice were obtained from The Jackson Laboratory Japan (Kanagawa, Japan). In the repeated-dose toxicity study, five-week-old male and female Crl:CD1 (ICR) mice were purchased from Charles River Laboratories Japan (Kanagawa, Japan). All mice were housed in individually ventilated cages under controlled environmental conditions (24°C ± 3°C, 50% ± 20% humidity, 12 h light/dark cycle). After subcutaneous tumor transplantation, mice were randomized into various groups using MiSTAT software (version 1.75; Mitsui Zosen Systems Research Inc., Tokyo, Japan), ensuring that the estimated mean tumor volume at baseline was within ±10% across groups. Body weights were measured using an analytical balance (GX-200; A&D Company, Ltd., Tokyo, Japan), and tumor diameters were measured with calipers. Tumor volume (mm^3^) was calculated using the formula: (short diameter)^2^ × long diameter/2. All efficacy, biodistribution, and toxicity studies were conducted by Hamri Co., Ltd. in accordance with Article 43 (“Reliability Criteria for Application Data”) of the Regulations for Enforcement of the Pharmaceuticals and Medical Devices Act of Japan. Animal experiments were also performed in compliance with the Law for the Humane Treatment and Management of Animals (Act No. 105 of 1973) and the Standards Relating to the Care and Management of Laboratory Animals and Relief of Pain (Ministry of the Environment Notification No. 88, 2006). All animal experiments were approved by the Institutional Animal Care and Use Committee of The University of Tokyo (approval numbers: 0320 and 0323). To minimize animal suffering, CVB3-WT, which is known to induce marked toxicity, was not used in the preclinical safety studies described herein.

### Virus construction

The full-length CVB3 cDNA plasmid was prepared as described previously.^15^ Recombinant CVB3 was generated using overlap extension PCR, with two tandem copies of the complementary sequences for miR-217 and miR-34a inserted into both the 5′ and 3′ UTRs of the CVB3 genome. All recombinant constructs were verified through Sanger sequencing.

### Virus production

*In vitro*-transcribed CVB3 RNA was transfected into H1299-T7 cells using Lipofectamine 2000 (Thermo Fisher Scientific). After 24 h, the culture supernatant was harvested and transferred to fresh H1299 cells. Following 5–6 h of incubation, the cells were collected with a cell scraper and subjected to three cycles of freeze-thawing in liquid nitrogen. The lysate was then centrifuged at 185 ×*g* for 15 min at 4°C, and the resulting supernatant was stored at −80°C as the virus stock.

### Virus titrations

H1299 cells were seeded into 96-well plates at a density of 5 × 10^3^ cells/well and incubated at 37°C for 7 h. Serial 10-fold dilutions of each virus were prepared in RPMI 1640 medium, and 50 μL of each dilution was added to each well. After 5 days of incubation at 37°C, cytopathic effects were examined under a stereomicroscope. Viral titers were calculated as TCID_50_ using the Reed-Muench method, as described previously.^13^

### *In vivo* experiment using H1299 cells

A total of 5 × 10^6^ H1299 cells were inoculated subcutaneously into the right flank of each mouse. When the tumor diameter reached 0.4–0.6 cm, the mice received five injections on alternate days of either CVB3-WT, CVB3-HP, CVB3-BHP (5 × 10^6^ TCID_50_/mouse), or saline. Tumor sizes and body weights were measured every other day and expressed as mean (SD). Mice were euthanized if the maximum tumor diameter exceeded 10 mm or if significant morbidity or weight loss occurred. For serum biochemical analyses, blood samples were collected the day after single or repeated (two doses) administration of the indicated viruses (CVB3-WT, CVB3-HP, or CVB3-BHP; 5 × 10^6^ TCID_50_/mouse) and analyzed using a fully automated chemistry analyzer (SPOTCHEM EZ SP-4430; ARKRAY, Kyoto, Japan). Pancreatic tissues were collected at the same time points for histopathological examination.

### Antitumor efficacy study

MDA-MB-468 cells (1 × 10^7^ cells/mouse) were transplanted subcutaneously into the right flank of each mouse. Once tumor diameters reached 0.4–0.6 cm, the mice were stratified and randomized to ensure a mean tumor volume within ±10% across groups. CVB3-BHP (1 × 10^5^, 1 × 10^6^, or 1 × 10^7^ TCID_50_/mouse) or saline was administered intratumorally on days 0, 3, 7, and 10. Tumor sizes and body weights were measured every other day and expressed as mean (SD). Mice were euthanized on day 28 after viral administration, and whole blood was collected for biochemical analysis.

### Biodistribution study

After acclimatization and stratification to ensure a mean body weight within ±10% across groups, mice were assigned to the control (saline), low-dose (1 × 10^6^ TCID_50_/mouse), or high-dose (1 × 10^7^ TCID_50_/mouse) groups and received repeated subcutaneous injections of CVB3-BHP on Days 0, 3, 7, and 10. On Days 11, 18, 25, and 46 after the first administration, blood was collected under anesthesia, followed by necropsy. Urine and feces samples were collected during necropsy. RNA was extracted from all samples, and CVB3 levels were quantified.

### Repeated-dose toxicity study

Following quarantine and acclimatization, mice were stratified by body weight (±10% range) and randomly assigned to a control group (saline) or toxicity groups (1 × 10^5^, 1 × 10^6^, 1 × 10^7^ TCID_50_/mouse). CVB3-BHP was administered on days 0, 3, 7, and 10, with necropsy performed on day 14. A separate recovery group (1 × 10^7^ TCID_50_/mouse) underwent necropsy on day 42. Urine samples were collected the day before necropsy, and food intake was recorded for the preceding 7 days. Clinical observations, body weight measurements, hematology, serum biochemistry, and histopathological examinations were conducted at necropsy in all groups.

### Biochemical analyses

Blood samples collected at necropsy in each *in vivo* experiment were analyzed using a fully automated hematology analyzer (XT-2000i; Sysmex Corporation, Kobe, Japan). Biochemical analysis of serum samples was performed with an automated analyzer (FUJI DRI-CHEM 7000VZ; FUJIFILM Medical Co., Ltd., Tokyo, Japan).

### RNA extraction and quantification using real-time PCR

Urinary RNA was extracted using the Ribospin vRD II kit (GeneAll Biotechnology, Seoul, South Korea), fecal RNA with the Stool Total RNA Purification Kit (Norgen Biotek Corp., Thorold, ON, Canada), and blood RNA using TRIzol LS Reagent (Thermo Fisher Scientific Inc.). Tissue RNA was purified using the RNeasy Mini Kit (QIAGEN, Hilden, Germany), with Buffer RLT volumes adjusted to tissue weight. Homogenization was performed using Biomashers II (Nippi, Inc., Tokyo, Japan), and RNA concentrations were measured using a DS-11+ spectrophotometer (DeNovix Inc., Wilmington, DE, USA). Real-time PCR targeting CVB3 and 18S rRNA (as an endogenous control) was conducted using the TaqMan probe method and TaqMan Fast Virus 1-Step Master Mix (Thermo Fisher Scientific) on a QuantStudio 3 Real-Time PCR System (Thermo Fisher Scientific). Absolute quantification of CVB3 was determined based on standard curves generated from reference standards.

### Histopathological examination

Organs and tissues excised at necropsy were fixed in 10% neutral-buffered formalin (FUJIFILM Wako Pure Chemical Corporation, Osaka, Japan), paraffin-embedded, sectioned, and stained with hematoxylin and eosin. For paired organs, the left side was examined unless gross abnormalities were present. Testes were fixed in formalin-sucrose-acetic acid solution, and eyeballs were fixed in 1% formaldehyde-2.5% glutaraldehyde buffer.

### Statistical analysis

All statistical analyses and figure generation were performed using GraphPad Prism version 10 (GraphPad software, San Diego, CA, USA) and Microsoft Excel 2019 (Microsoft Corporation, Redmond, WA, USA). Group comparisons were conducted using one-way analysis of variance or Dunnett’s multiple comparison test, with additional two-tailed Student’s *t* tests when required. Statistical significance was set at *p* < 0.05. Survival curves were generated using the Kaplan-Meier method, and comparisons were made using the log rank test. No statistical analyses were performed for clinical signs, food consumption, urinalysis, gross necropsy, or histopathological findings.

## Data and code availability

Data and materials described in this article are available upon reasonable request to the corresponding author.

## Acknowledgments

This work was supported by 10.13039/100009619AMED under grant no. JP20lm0203139.

## Author contributions

Y.S. drafted the original manuscript and prepared the figures. Y.S. and S.M. contributed equally to data analysis, manuscript revision, and critical editing. K.T. was responsible for funding acquisition and, together with M.M., supervised the overall study, provided conceptual guidance, and contributed to project administration. M.S., S.I., Y.K., and Y.M. provided technical assistance and supported experimental procedures. F.Y. and K.E. oversaw the study and provided critical supervision and guidance throughout the project.

## Declaration of interests

K.T. is a shareholder of NPT Co., Ltd., which also holds a patent related to CVB3-BHP.

## Supplemental information

Document S1. Figure S1

Table S1. Urinalysis, food intake, and ophthalmologic assessment in male and female mice following repeated subcutaneous administration of CVB3-BHP

Table S2. Histopathological findings in male and female mice from the toxicity groups

Table S3. Histopathological findings in male and female mice from the recovery groups

Document S2. Article plus supplemental information
