# Supplementary material for: Comprehensive preclinical evaluation of second-generation miRNA-regulated Coxsackievirus B3-BHP: Potent antitumor efficacy and safety
Source: Mol Ther Oncol. 2026 Aug 21;34(3):201332. doi: 10.1016/j.omton.2026.201332 (PMC13571354; doi:10.1016/j.omton.2026.201332)
Supplement: Supplementary file 1 [file mmc1.pdf]

**OMTON, Volume *34***

## Supplemental information

# Comprehensive preclinical evaluation of second-generation miRNA-regulated Coxsackievirus B3-BHP: Potent antitumor efficacy and safety

**Yuki Sakashita, Shohei Miyamoto, Miyako Sagara, Shun Ito, Yukiko Kobayashi, Yoshio Momoda, Fumiaki Yano, Ken Eto, Kenzaburo Tani, and Mutsunori Murahashi**

A

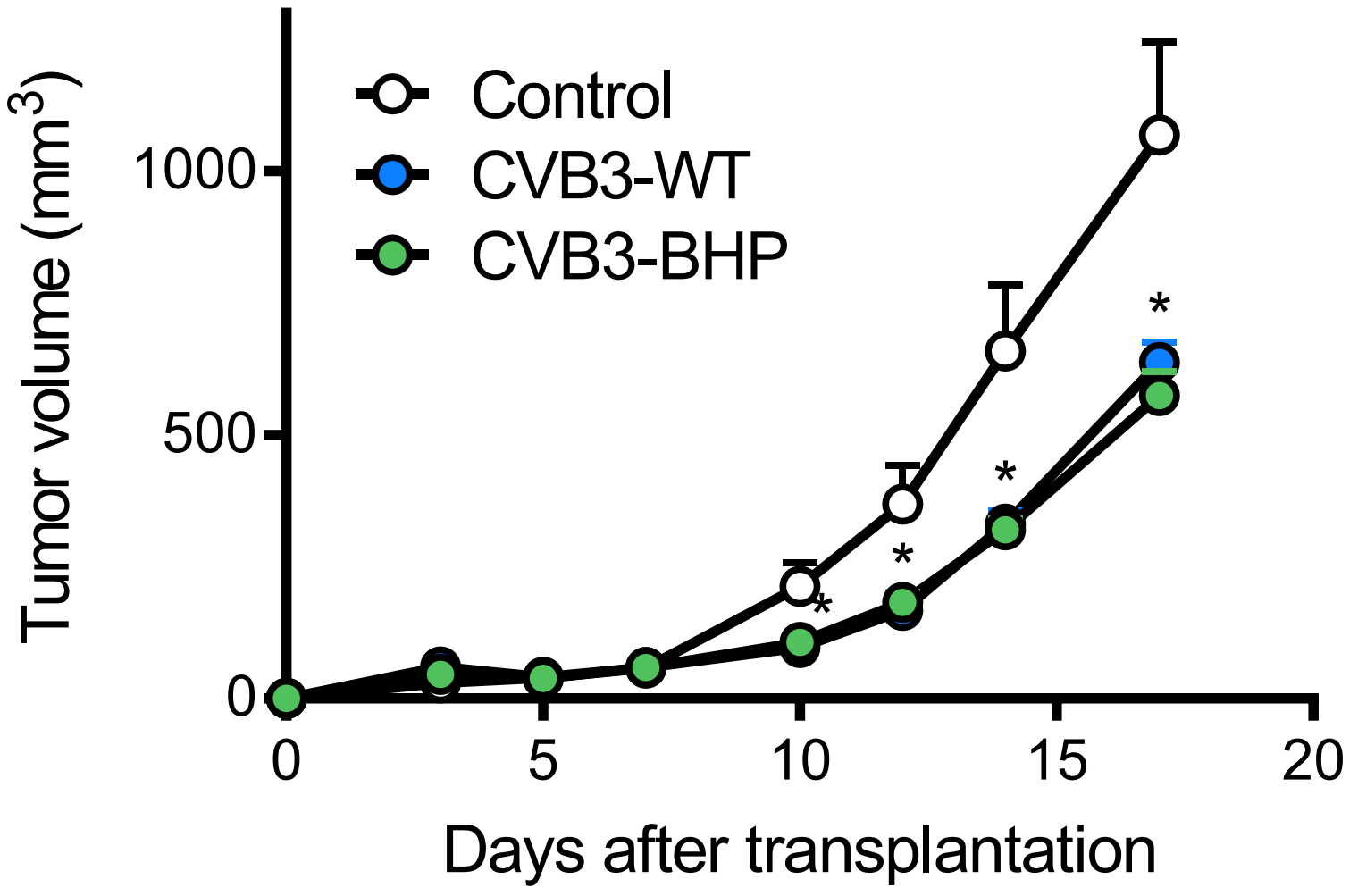


B

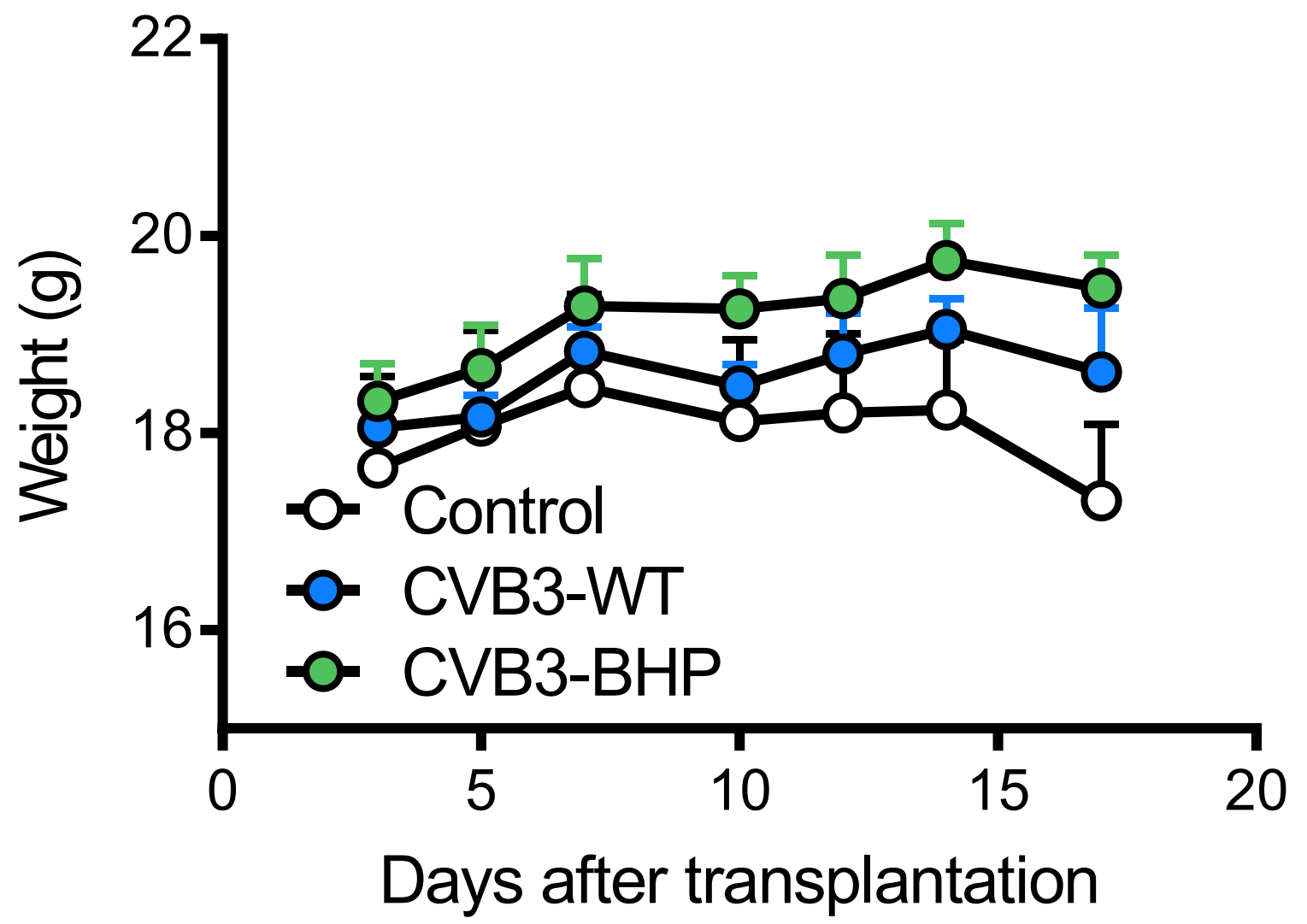


**Figure S1.** Antitumor efficacy of CVB3-WT and CVB3-BHP in an immunocompetent TC-1 syngeneic tumor model. Antitumor efficacy was evaluated using a TC-1 syngeneic tumor model in immunocompetent C57BL/6 mice. TC-1 cells ($5 \times 10^5$ cells in 100 µL PBS) were subcutaneously implanted into the right flank on Day 0. Mice were randomly assigned to the control, CVB3-WT, or CVB3-BHP group (n = 6 per group). Intratumoral administration of CVB3-WT or CVB3-BHP ($5 \times 10^6$ $TCID_{50}$ per injection) was performed on Days 7, 10, 14, and 17. The experiment was terminated on Day 17 because tumors in the control group exceeded 1,000 mm³.

(A) Tumor growth curves. Tumor volume was measured throughout the study. Data are presented as mean (SD). Statistical analysis was performed using one-way ANOVA.

$p < 0.05$ versus the control group for both CVB3-WT and CVB3-BHP at the corresponding time points (Days 10, 12, 14, and 17).

**(B)** Body weight changes during treatment. Body weight was monitored throughout the study. Data are presented as mean (SD).

***Abbreviations***: CVB3-BHP, second-generation recombinant Coxsackievirus B3; CVB3-WT, wild-type Coxsackievirus B3; PBS, phosphate-buffered saline; $TCID_{50}$, tissue culture infectious dose 50%.

**Table S1. Urinalysis, food intake, and ophthalmologic assessment in male and female mice following repeated subcutaneous administration of CVB3-BHP.**
Urinalysis, food intake monitoring, and ophthalmologic examinations were performed in male and female mice after subcutaneous administration of CVB3-BHP ($1.0 \times 10^5$, $1.0 \times 10^6$, or $1.0 \times 10^7$ $TCID_{50}$/mouse) on Days 0, 3, 7, and 10.
**(A)** Urinalysis: Urine samples were collected on Day 13 for the toxicity groups (n = 6–10 per sex per dose, depending on sample availability) and Day 41 for the recovery groups (n = 6 per group).
**(B)** Food intake: Food consumption was measured daily for 7 consecutive days prior to necropsy — Days 7–14 for the toxicity groups (n = 10 per group) and Days 35–42 for the recovery groups (n = 6 per group).
**(C)** Ophthalmologic examinations: Examinations were performed under isoflurane anesthesia using a portable slit lamp and a handheld fundus camera on Day 13 for the toxicity groups (n = 10 per group) and Day 37 for the recovery groups (n = 6 per group).
***Abbreviations***: CVB3-BHP, second-generation recombinant Coxsackievirus B3; $TCID_{50}$, tissue culture infectious dose 50%

Due to the large number of tissues and evaluated parameters, this table is provided as a separate Excel file (Table S1.xlsx).

**Table S2. Histopathological findings in male and female mice from the toxicity groups.**
Histopathological analysis was conducted on tissues collected from male and female mice (n = 10 per group) on Day 14, following repeated subcutaneous administration of CVB3-BHP ($1.0 \times 10^5$, $1.0 \times 10^6$, or $1.0 \times 10^7$ $TCID_{50}$/mouse on Days 0, 3, 7, and 10).
**(A)** Representative findings from male mice.
**(B)** Representative findings from female mice.
Abbreviations: CVB3-BHP, second-generation recombinant Coxsackievirus B3; $TCID_{50}$, tissue culture infectious dose 50%

Due to the large number of tissues and evaluated parameters, this table is provided as a separate Excel file (Table S2.xlsx).

**Table S3. Histopathological findings in male and female mice from the recovery groups.**
Histopathological analysis was conducted on tissues collected from male and female mice (n = 6 per group) on Day 42, following repeated subcutaneous administration of CVB3-BHP ($1.0 \times 10^7$ $TCID_{50}$/mouse on Days 0, 3, 7, and 10).
**(A)** Representative findings from male mice.
**(B)** Representative findings from female mice.
Abbreviations: CVB3-BHP, second-generation recombinant Coxsackievirus B3; $TCID_{50}$, tissue culture infectious dose 50%

Due to the large number of tissues and evaluated parameters, this table is provided as a separate Excel file (Table S3.xlsx).
